# Supplementary material for: Incidence and Risk Factors of White Matter Lesions in Moderate and Late Preterm Infants
Source: Diagnostics (Basel). 2025 Apr 1;15(7):881. doi: 10.3390/diagnostics15070881 (PMC11988739; doi:10.3390/diagnostics15070881)
Supplement: Supplementary file 1 [file diagnostics-15-00881-s001.zip › diagnostics-3485532-supplementary.pdf]

Supplementary Table S1. Dependence of punctate white matter lesions of six or more lesions on clinical variables

	OR	95% CI		p
		Lower	Upper	
**Univariate analysis**				
Maternal and antenatal variables				
Vaginal delivery	3.657	0.813	16.454	0.091
PROM	7.765	1.776	33.942	0.006
Antenatal steroids	0.604	0.118	3.097	0.545
Clinical chorioamnionitis	N/A			N/A
Threatened preterm labour	0.532	0.123	2.305	0.399
Foetal distress	N/A			N/A
Placenta praevia	N/A			N/A
Hypertensive disorders in pregnancy	N/A			N/A
Variables at birth				
Male sex	3.574	0.698	18.284	0.126
Gestational age (week)	1.288	0.704	2.357	0.411
Body weight at birth (per 100g)	1.243	1.010	1.528	0.040
Body weight z-score	1.851	0.877	3.905	0.106
Apgar score				
1min.	0.782	0.549	1.114	0.173
5min.	0.548	0.353	0.853	0.008
Cord blood				
pH (per 0.1)	0.805	0.231	2.812	0.734
Base excess (mmol/L)	0.812	0.653	1.011	0.062
Need of resuscitation	8.803	1.057	73.345	0.044
Need for intubation	8.338	1.001	69.463	0.050
Duration of intubation	1.236	0.916	1.667	0.166
Any treatment for PDA	N/A			N/A
Inotropes use	N/A			N/A
Transfusion	N/A			N/A
Postnatal steroid	N/A			N/A
MRI scan				
Postmenstrual age at scan (week)	0.474	0.170	1.321	0.154
Postnatal age at scan (days)	0.886	0.785	0.999	0.048

Abbreviation: CI, confidence interval; OR, odds ratio; PROM, premature rupture of the membrane; PDA, patent ductus arteriosus; MRI, magnetic resonance imaging

Supplementary Table S2. Dependence of grey matter lesions on clinical variables

	OR	95% CI		p
		Lower	Upper	
**Univariate analysis**				
Maternal and antenatal variables				
Vaginal delivery	0.492	0.049	4.957	0.547
PROM	N/A			N/A
Antenatal steroids	N/A			N/A
Clinical chorioamnionitis	N/A			N/A
Threatened preterm labour	0.295	0.030	2.905	0.296
Foetal distress	13.900	1.767	109.314	0.012
Placenta praevia	N/A			N/A
Hypertensive disorders in pregnancy	2.150	0.213	21.696	0.516
Variables at birth				
Male sex	1.191	0.163	8.682	0.863
Gestational age (week)	0.970	0.446	2.109	0.939
Body weight at birth (per 100g)	1.000	0.767	1.304	0.998
Body weight z-score	0.845	0.348	2.050	0.710
Apgar score				
1min.	0.592	0.411	0.854	0.005
5min.	0.504	0.304	0.835	0.008
Cord blood				
pH (per 0.1)	0.280	0.121	0.651	0.003
Base excess (mmol/L)	0.726	0.588	0.897	0.003
Need of resuscitation	3.773	0.384	37.112	0.255
Need for intubation	3.574	0.363	35.148	0.275
Duration of intubation	1.455	1.028	2.060	0.034
Any treatment for PDA	N/A			N/A
Inotropes use	86.400	7.588	983.835	< 0.001
Transfusion	6.762	0.621	73.575	0.117
Postnatal steroid	49.333	2.461	989.130	0.011
MRI scan				
Postmenstrual age at scan (week)	0.663	0.209	2.104	0.485
Postnatal age at scan (days)	0.973	0.873	1.084	0.615

Abbreviation: CI, confidence interval; OR, odds ratio; PROM, premature rupture of the membrane; PDA, patent ductus arteriosus; MRI, magnetic resonance imaging

Supplementary Table S3. Dependence of brain lesions on clinical variables

	No lesion n=149	Brain lesion n=46	OR	95% CI		p
				Lower	Upper	
**Univariate analysis**						
Maternal and antenatal variables						
Vaginal delivery	21 (14.1)	15 (32.6)	2.949	1.366	6.370	0.006
PROM	10 (6.7)	1 (2.2)	0.309	0.038	2.480	0.269
Antenatal steroids	53 (35.6)	20 (43.5)	1.393	0.711	2.730	0.334
Clinical chorioamnionitis	5 (3.4)	4 (9.3)	2.892	0.741	11.289	0.126
Threatened preterm labour	79 (53.0)	26 (56.5)	1.152	0.592	2.242	0.677
Foetal distress	10 (6.7)	2 (4.3)	0.632	0.133	2.993	0.563
Placenta praevia	6 (4.0)	0 (0.0)	N/A			N/A
Hypertensive disorders in pregnancy	20 (13.4)	2 (4.3)	0.293	0.066	1.305	0.107
Variables at birth						
Male sex	68 (45.6)	29 (63.0)	2.032	1.029	4.011	0.041
Gestational age (week)	35.0 (1.3)	34.9 (1.1)	0.954	0.729	1.248	0.731
Body weight at birth (per 100g)	2067 (371)	2208 (387)	1.105	1.010	1.209	0.029
Body weight z-score	-0.60 (1.10)	-0.19 (0.92)	1.465	1.052	2.040	0.024
Apgar score						
1min.	8 [8-8]	8 [7-8]	0.856	0.710	1.031	0.100
5min.	9 [8-9]	9 [8-9]	0.731	0.560	0.955	0.022
Cord blood						
pH (per 0.1)	7.318 (0.051)	7.293 (0.125)	0.678	0.440	1.044	0.078
Base excess (mmol/L)	-1.7 (2.6)	-3.3 (5.0)	0.877	0.788	0.976	0.016

Need of resuscitation	66 (44.3)	28 (60.9)	1.956	0.996	3.841	0.051
Need for intubation	68 (45.6)	31 (67.4)	2.462	1.228	4.936	0.011
Duration of intubation	1.2 (1.8)	2.2 (3.5)	1.185	1.022	1.373	0.024
Any treatment for PDA	7 (4.7)	0 (0.0)	N/A			N/A
Inotropes use	5 (3.4)	6 (13.0)	4.320	1.253	14.890	0.020
Transfusion	7 (4.7)	3 (6.5)	1.415	0.351	5.710	0.626
Postnatal steroid	1 (0.7)	4 (8.7)	14.095	1.534	129.512	0.019
MRI scan						
Postmenstrual age at scan (weeks)	37.6 (1.3)	37.2 (1.0)	0.708	0.492	1.018	0.062
Postnatal age at scan (days)	18.5 (10.9)	16.2 (9.8)	0.977	0.943	1.013	0.207
**Multivariate analysis**						
Gestational age (week)			1.025	0.768	1.366	0.868
Male sex (male)			1.788	0.868	3.683	0.115
Vaginal delivery			2.382	1.234	6.500	0.014
Body weight at birth (z-score)			1.454	1.012	2.089	0.043
Need for intubation			2.707	1.293	5.668	0.008

Abbreviation: CI, confidence interval; OR, odds ratio; PROM, premature rupture of the membrane; PDA, patent ductus arteriosus; MRI, magnetic resonance imaging
